# Relevance of Ferroptosis to Cardiotoxicity Caused by Anthracyclines: Mechanisms to Target Treatments

**DOI:** 10.3389/fcvm.2022.896792

**Published:** 2022-06-13

**Authors:** Guoxia Zhang, Chao Yuan, Xin Su, Jianzhen Zhang, Priyanka Gokulnath, Gururaja Vulugundam, Guoping Li, Xinyu Yang, Na An, Can Liu, Wanli Sun, Hengwen Chen, Min Wu, Shipeng Sun, Yanwei Xing

**Affiliations:** ^1^Guang’anmen Hospital, China Academy of Chinese Medical Sciences, Beijing, China; ^2^Dezhou Second People’s Hospital, Dezhou, China; ^3^Dongzhimen Hospital, Beijing University of Chinese Medicine, Beijing, China; ^4^Cardiovascular Division of the Massachusetts General Hospital and Harvard Medical School, Boston, MA, United States; ^5^Institute of Biochemistry and Cellular Biology, National Research Council of Italy, Naples, Italy; ^6^Fangshan Hospital, Beijing University of Chinese Medicine, Beijing, China

**Keywords:** ferroptosis, doxorubicin, iron, treatment, mechanism, cardiotoxicity

## Abstract

Anthracyclines (ANTs) are a class of anticancer drugs widely used in oncology. However, the clinical application of ANTs is limited by their cardiotoxicity. The mechanisms underlying ANTs-induced cardiotoxicity (AIC) are complicated and involve oxidative stress, inflammation, topoisomerase 2β inhibition, pyroptosis, immunometabolism, autophagy, apoptosis, ferroptosis, etc. Ferroptosis is a new form of regulated cell death (RCD) proposed in 2012, characterized by iron-dependent accumulation of reactive oxygen species (ROS) and lipid peroxidation. An increasing number of studies have found that ferroptosis plays a vital role in the development of AIC. Therefore, we aimed to elaborate on ferroptosis in AIC, especially by doxorubicin (DOX). We first summarize the mechanisms of ferroptosis in terms of oxidation and anti-oxidation systems. Then, we discuss the mechanisms related to ferroptosis caused by DOX, particularly from the perspective of iron metabolism of cardiomyocytes. We also present our research on the prevention and treatment of AIC based on ferroptosis. Finally, we enumerate our views on the development of drugs targeting ferroptosis in this emerging field.

## Introduction

With the advancements in medical technology, while the survival time of cancer patients has been prolonged, cardiovascular toxicity has become one of the most severe complications of cancer treatment (1, 2). Studies have shown that cancer survivors are at an eight-times higher risk of developing cardiovascular disease (CVD) than the general population (3). Anthracyclines (ANTs) are a class of chemotherapy drugs commonly used in clinical practice that significantly improve the survival rate of patients. However, the use of ANTs is restricted due to their cardiotoxic effects (2, 4). The incidence of left ventricular dysfunction, which is up to 48%, is positively correlated with dose (2). In some cancer survivors, the death rate of CVDs even exceeds that of their primary cancers (5). Therefore, it is necessary to explore the mechanism of cardiotoxicity caused by cancer therapy. The mechanisms of ANTs-induced cardiotoxicity (AIC) involve oxidative stress, inflammation, topoisomerase 2β inhibition, pyroptosis, immunometabolism, autophagy, apoptosis, etc. (6–8). Besides, in recent years, more and more studies have shown that ferroptosis plays a vital role in AIC (9, 10). Inhibiting the ferroptosis of cardiomyocytes can reduce AIC, which may be a novel prevention and treatment strategy in cardio-oncology.

Ferroptosis is a new form of regulated cell death (RCD) different from apoptosis, necrosis, necroptosis, pyroptosis, and autophagy. It is characterized by iron overload and reactive oxygen species (ROS) accumulation, resulting in lipid peroxidation of cell membranes (11, 12). A few decades ago, it was demonstrated that glutamate could inhibit the uptake of cystine, leading to a decrease in glutathione (GSH) levels within cells, thereby causing oxidative death of cells, and termed this process as “oxytosis” (13, 14). We believe that doxorubicin (DOX) can induce ferroptosis in cardiomyocytes through the following mechanisms: firstly, by regulating iron homeostasis-related proteins and iron-responsive elements (IREs)/iron regulatory proteins (IRPs), leading to increased iron levels in cardiomyocytes; secondly, DOX can increase ROS, thereby causing cell membrane lipid peroxidation. The nuclear factor (erythroid-derived 2)-like 2 (Nrf2) signaling pathway plays an important role. As the central organelle for ROS generation and the site where iron accumulation may occur, mitochondria are crucial for developing doxorubicin-induced cardiomyopathy (DIC). Further, we summarize the current treatments to prevent and treat AIC by inhibiting the ferroptosis of cardiomyocytes. Finally, we provide our future perspectives on this emerging field.

## Mechanisms of Ferroptosis

**FIGURE 1:**
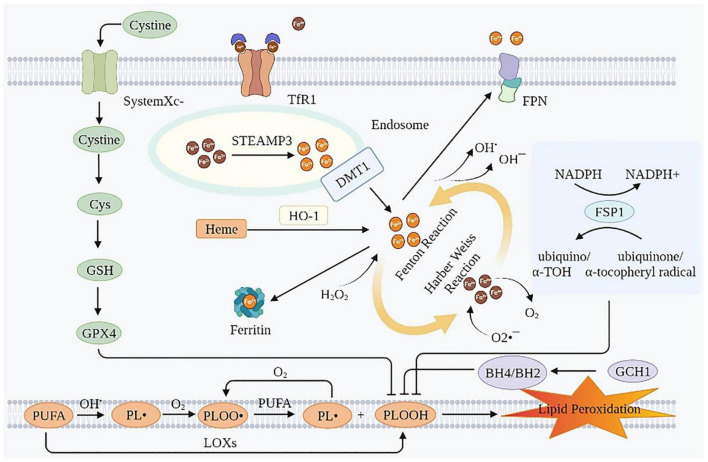
Mechanisms of ferroptosis. Ferroptosis is essentially an iron-dependent lipid peroxidation. Intracellular iron overload is a necessary condition for ferroptosis, and lipid peroxidation is the presentation form of ferroptosis. TfR1, transferrin receptor 1; FPN, ferroportin; Cys, cysteine; GSH, glutathione; GPX4, glutathione peroxidase 4; STEAP3, six-transmembrane epithelial antigen of the prostate 3; DMT1, divalent metal transporter 1; HO-1, heme oxygenase 1; NADPH, nicotinamide adenine dinucleotide phosphate; FSP1, ferroptosis suppressor protein 1; PUFA, polyunsaturated fatty acids; GCH1, GTP cyclohydrolase-1; BH4/BH2, tetrahydrobiopterin/dihydrobiopterin; LOXs, lipoxygenases; PL^•^, phospholipid radical; PLOO^•^, phospholipid peroxyl radical; PLOOH, phospholipid hydroperoxide; α-TOH, α-tocopherol.

Ferroptosis is an iron-dependent lipid peroxidation induced novel RCD, caused by redox imbalances between the oxidant and antioxidant systems. Antioxidant systems include the Cyst(e)ine-GSH-glutathione peroxidase 4 (GPX4) pathway, the ferroptosis suppressor protein 1 (FSP1)-coenzyme Q10 (CoQ10)-nicotinamide adenine dinucleotide phosphate (NADPH) pathway, the GTP cyclohydrolase-1 (GCH1)-tetrahydrobiopterin (BH4) pathway, etc. (15, 16). Intracellular iron overload is a necessary condition for ferroptosis. Therefore, lipid peroxidation is the most common cause of ferroptosis (15) (Figure 1).

### Oxidation System

#### Iron Overload

Iron overload is a prerequisite of ferroptosis. The erastin-induced ferroptosis was inhibited by deferoxamine (DFO, an iron chelator), evidenced by increased cell viability and decreased lipid ROS production in HT-1080 cells. In contrast, the erastin-induced ferroptosis was triggered by incubation with three different exogenous iron supplements (11). In intestinal ischemia/reperfusion-induced acute lung injury model of C57BL/6 mice, the injection of Fe (15 mg/kg) aggravated lung injury and pulmonary edema, while the injection of ferrostatin-1 (Fer-1, 5 mg/kg) rescued this injury (17). Iron transport involves import, storage, and export (18, 19). Circulating iron exists in the form of ferric iron (Fe^3+^) by binding to transferrin (Tf). Fe^3+^ enters the endosome through membrane protein transferrin receptor 1 (TfR1). Then, Fe^3+^ is reduced to ferrous iron (Fe^2+^) by the iron reductase activity of the six-transmembrane epithelial antigen of the prostate 3. The divalent metal transporter 1 (DMT1, also known as SLC11A2) releases Fe^2+^ from the endosome into the cytoplasm. While part of the Fe^2+^ in the cytoplasm is stored as ferritin, part is oxidized to Fe^3+^ and transported outside the cell by the membrane protein iron transporter ferroportin (FPN, an iron efflux pump, also known as SLC11A3), and the rest is stored in the labile iron pool (LIP) of the cytoplasm or mitochondria (20). The iron in LIP spontaneously undergoes redox reactions, namely, Fenton and Harber Weiss reactions, to generate ROS, which in turn leads to lipid peroxidation (21). Moreover, iron and iron derivatives, such as heme or [Fe-S] clusters, also affect ferroptosis as they act on the active centers of ROS producing enzymes, such as lipoxygenase (LOX), cytochrome P450, NADPH oxidase and so on (22). Therefore, iron overload is essential for ferroptosis. Maintaining the LIP within a relatively narrow concentration range is crucial for preventing ferroptosis. Using DFO could prevent cell death caused by erastin and RSL3 (11, 23).

In this process, the core negative regulators of ferroptosis are ferritin heavy chain (FTH) (24–27) and FPN (28), and the core positive regulators of ferroptosis are Tf (29–31), TfR1 (24, 26, 31–33), and DMT1 (24, 34, 35). These iron homeostasis proteins involved in iron uptake, storage, utilization, and efflux from cells are regulated by IREs/IRPs (36, 37). In 1-methyl-4-phenyl-1,2,3,6-tetrahydropyridine-induced PD mice models, apoferritin inhibited ferroptosis by downregulating the iron importers DMT1 and FSP1, and conversely upregulating long-chain acyl-CoA synthetase 4 (38). The lipopolysaccharide then increased the expression of nuclear receptor co-activator 4, which directly interacted with ferritin and degraded ferritin in a ferritin phagocytosis-dependent manner. It then released a large amount of iron (39). In addition, heme oxygenase 1 (HO-1) mediates the release of free iron from heme, resulting in the accumulation of Fe^2+^ in LIP, which also exacerbates ferroptosis (40, 41).

#### Lipid Peroxidation

Fe^2+^ in LIP can spontaneously undergo redox reactions to produce ROS, including both Fenton and Harber-Weiss reactions. The chemical equations are as follows (42):


$$\mathrm{The}\,\mathrm{Fenton}\,\mathrm{reaction}\,\mathrm{is}:{\mathrm{Fe}}^{2+}+{\text{H}}_{2}\,{\text{O}}_{2}\to {\text{Fe}}^{3+}+{\text{OH}}^{•}+{\text{OH}}^{-}.$$

$$\mathrm{The}\,\mathrm{Harber}-\mathrm{Weiss}\,\mathrm{reaction}\,\mathrm{is}:{\mathrm{Fe}}^{3+}+{\text{O}}_{2}^{•-}\to {\text{Fe}}^{2+}+{\text{O}}_{2}.$$

$$\mathrm{The}\,\mathrm{overall}\,\mathrm{reaction}\,\mathrm{ROS}\,\mathrm{is}:O_{2}^{•-}+{\text{H}}_{2}\,{\text{O}}_{2}\to {\text{OH}}^{•}+{\text{OH}}^{-}+{\text{O}}_{2}.$$

The electron transport system of mitochondria is the primary source of H_2_O_2_ and active oxygen (O_2_^•–^) (43). ROS, generated by the above mechanism, causes damage to the biomembrane in two ways. One is an enzyme-independent way, that is: Firstly, the hydroxyl radical (OH^•^) combines with polyunsaturated fatty acids (PUFA) on the biomembrane to generate the phospholipid radical (PL^•^). Secondly, PL^•^ reacts with O_2_ generating a phospholipid peroxyl radical (PLOO^•^). Thirdly, PLOO^•^ reacts with PUFA to generate phospholipid hydroperoxide (PLOOH) and PL^•^, which can react again with O_2_, forming a vicious circle. The other is the enzymatic way, that is, PUFA generates PLOOH under the action of LOXs. However, the detailed mechanism of PLOOH resulting in ferroptotic lipid peroxidative cell death remains obscure. Continued oxidation and consumption of PUFA may alter the structure of lipid pores, ultimately leading to compromised membrane integrity. In addition, PLOOH may be decomposed into active toxic aldehydes, such as 4-hydroxy-2-nonenal or malondialdehyde (MDA), causing cytotoxic effects (44). The hallmark of ferroptosis is the iron-dependent accumulation of lipid hydroperoxides to cell-lethal levels, especially peroxidized phosphatidylethanolamine (PEox). However, only a few studies detected and quantified these directly. In heart transplantation mice models, hydroperoxy-arachidonoyl-phosphatidylethanolamine (HOO-C20:4/C18:0-PE) was elevated and the resulting ferroptosis triggered early inflammation by recruiting neutrophils. In IRI mice models, the abundance of several hydroxyeicosatetraenoic acids (HETE) (such as 5-HETE, 11-HETE, 12-HETE, and 15-HETE) and epoxyeicosatrienoic acid species were increased (45). Besides, in RSL3-induced ferroptosis in H9C2 cardiomyocytes, *via* LC/MS, three significant species of hydroperoxy-PE were found to be up-regulated, namely, PE(36:4)-OOH, PE(38:4)-OOH, and PE(40:4)-OOH (46). Sparvero et al. used gas cluster ion beam secondary ion mass spectrometry imaging with a 70 keV (H_2_O) (_*n*_) (+) (n > 28000) cluster ion beam to visualize them at the single-cell and subcellular levels (47). The ferroptosis inhibitor, Fer-1 inhibited ferroptosis by reducing PLOOH.

### Anti-oxidation System

#### The Cyst(e)ine-Glutathione-Glutathione Peroxidase 4 Pathway

The cyst(e)ine-GSH-GPX4 pathway is considered the canonical pathway for restricting ferroptosis. System Xc^–^, a heterodimeric 12-pass transmembrane cystine–glutamate anti-porter, consists of a xCT light chain (also known as SLC7A11) that mediates cystine transport specificity, and a 4F2 heavy chain (also known as SLC3A2) (48), plays an important role in this process. SLC7A11 transports extracellular cystine, which is rapidly reduced to cysteine (Cys) by an NADPH-consuming reduction process. Cys participates in the production of GSH, a fundamental component of GPX4 (49). GPX4 is the primary inhibitor of ferroptosis. It can prevent lipid peroxidation by reducing PLOOH to non-toxic phospholipid alcohols (49). Cardiac impairments were ameliorated in GPX4 Tg mice and exacerbated in GPX4 heterodeletion mice. In cultured cardiomyocytes, GPX4 overexpression prevented DOX-induced ferroptosis (50). Surprisingly, a recent study showed that in non-small-cell lung cancer cell lines, cystine starvation induces an unexpected accumulation of γ-glutamyl-peptides under the influence of glutamate-cysteine ligase catalytic subunit, which limits the accumulation of glutamate, thereby protecting against ferroptosis (51). In addition, methionine can be used as one of the sources of intracellular cystine through the trans-sulfuration pathway (49). GPX4 is a selenoenzyme and its biosynthesis relies on the co-translational incorporation of selenocysteine (49). Selenium augments GPX4 and other genes by enhancing adaptive transcription factors TFAP2c and Sp1 to protect the cells from ferroptosis (52).

#### The Ferroptosis Suppressor Protein 1-Coenzyme Q10-Nicotinamide Adenine Dinucleotide Phosphate Pathway

The FSP1-CoQ10-NADPH pathway exists as a GPX4-independent one. FSP1 catalyzes the transformation of CoQ10 into ubiquinol, which is an excellent radical-trapping antioxidant in phospholipids and lipoproteins (53–55). Furthermore, the pathway can reduce oxidized α-tocopheryl radical to its non-radical form, increasing antioxidant capacity (56). The MDM2-MDMX complex is a negative regulator of FSP1. It changes the activity of PPARα, resulting in a decrease in the level of FSP1 protein and an increase in the level of CoQ10 (57). MiR-4443, whose target gene is METLL3, inhibited FSP1-mediated ferroptosis induced by cisplatin treatment *in vitro* and enhanced tumor growth *in vivo* (58).

#### The GTP-GTP Cyclohydrolase-1-Tetrahydrobiopterin Signaling Pathway

The GTP-GCH1-BH4 pathway is also not dependent on GPX4. BH4/dihydrobiopterin synthesis by GCH1-expressing cells caused lipid remodeling, suppressing ferroptosis by selectively preventing depletion of phospholipids with two polyunsaturated fatty acyl tails (59). Using a co-culture model system, iNOS/NO (^•^) in M1 macrophages has been confirmed to inhibit the effect of NO (^•^) on epithelial cells by inhibiting phospholipid peroxidation, especially the generation of 15-HpETE-PE signal that promotes ferroptosis. It is an intercellular mechanism that distantly prevents the ferroptosis of epithelial cells stimulated by *Pseudomonas aeruginosa* (60).

Nuclear factor (erythroid-derived 2)-like 2 signaling is implicated in many molecular aspects of ferroptosis, including glutathione homeostasis, mitochondrial function, and lipid metabolism (61, 62). The Nrf2-Focad-Fak signaling pathway is closely related to ferroptosis caused by Cys deprivation. In non-small-cell lung carcinoma, brusatol (an Nrf2 inhibitor) was added based on ferroptosis inducer erastin or RSL3. The therapeutic effect based on ferroptosis was better than single treatment *in vivo* and *in vitro* (63). In immunocompetent mice and humanized mice, ZVI-NP, a dual-functional nanomedicine, enhanced the degradation of Nrf2 by GSK3/β-TrCP through AMP-activated protein kinase (AMPK)/rapamycin activation, leading to cancer-specific ferroptosis of lung cancer cells (64).

#### The Other Antioxidant Elements

The antioxidant system of the heart is very complex. In addition to the above three major systems, the antioxidant system of the heart also includes some other elements that inhibit ferroptosis. O_2_^•–^, OH^•^, OH^–^, H_2_O_2_, PL^•^, PLOO^•^, PLOOH, ROS, etc. play important roles in the occurrence and development of ferroptosis. Superoxide dismutase (SOD) and superoxide reductases can reduce O_2_^•–^ to H_2_O_2_. Catalase catalyzes H_2_O_2_ to water and O_2_. Water-soluble ascorbic acid (vitamin C), lipid-soluble vitamin E or α-tocopherol (α-TOH), and lipoic acid can reduce lipid hydroperoxide production and peroxyl radicals. Besides, ascorbic acid increases the vitamin E content by reducing vitamin E semiquinone (65). The thioredoxin system, consisting of the thioredoxin (Trx) and thioredoxin reductase (TrxR), is also an important antioxidant system (66). Ferroptocide causes an accumulation of lipid peroxidation by inhibiting this system, thereby inducing ferroptosis (67). TrxR and NADPH reduce the active site disulfide in Trx. Under the combination of Trx and TrxR, peroxides, including lipid hydroperoxides and H_2_O_2_ were observed to be reduced effectively. In addition, there are several crosstalks between these antioxidants. For example, the thioredoxin system promotes the regeneration of certain antioxidants. It reduces ascorbyl free radical to ascorbic acid and turns GSSG to GSH. The thioredoxin system increases the content of ascorbic acid by reducing dehydroascorbic acid (65).

## Ferroptosis and Anthracycline-Induced Cardiotoxicity

**FIGURE 2:**
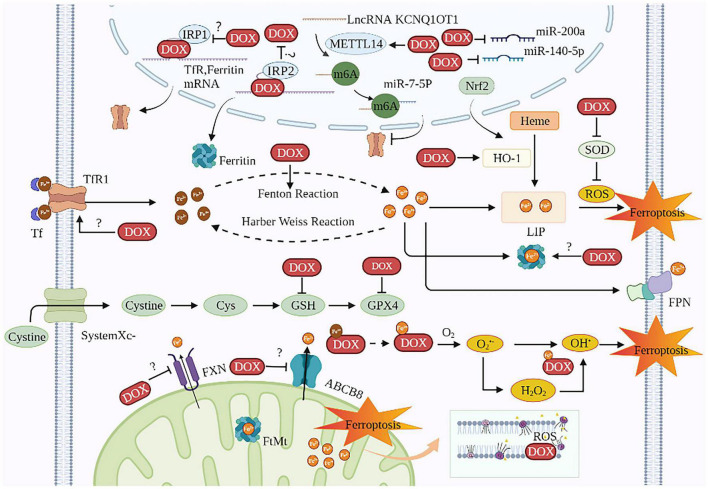
The mechanisms of DIC based on ferroptosis. DOX induces ferroptosis in cardiomyocytes involves two major mechanisms: one is to disrupt iron homeostasis and the other is to promote lipid peroxidation. The targets of DOX on iron disorder are Tf, ferritin, HO-1, FXN, ABCB8, IRE, IRP, and KCNQ1OT1m6A. The targets of DOX for lipid peroxidation are ROS, SOD, GPX4, and GSH. The site of iron death in cardiomyocytes is probably the mitochondria. TfR1, transferrin receptor 1; Tf, transferrin; DOX, doxorubicin; IRP, iron regulatory protein; METTL14, methyltransferase-like 14; Nrf2, nuclear factor (erythroid-derived 2)-like 2; LIP, labile iron pool; HO-1, heme oxygenase 1; ROS, reactive oxygen species; SOD, superoxide dismutase; Cys, cysteine; GSH, glutathione; GPX4, glutathione peroxidase 4; FPN, ferroportin; FXN, frataxin; ABCB8, ABC protein-B8; O_2_, oxygen; O_2_^•–^, active oxygen; FtMt, mitochondrial ferritin; CL, cardiolipin; OH^•^, hydroxyl radical.

Doxorubicin is one of the most cardiotoxic anticancer agents. Current studies on DIC based on ferroptosis mainly focus on DOX. DOX’s anti-cancer activity is primarily mediated by DNA intercalation and inhibition of the topoisomerase II enzyme in rapidly proliferating tumors. However, DOX causes cumulative and dose-dependent cardiotoxicity, resulting in increased mortality risks among cancer patients and thus limits its wide clinical applications (68). Ferroptosis is involved in DIC both *in vivo* and *in vitro* (50, 69–72). The survival rate of rats was markedly elevated with the ferroptosis inhibitor Fer-1 than with apoptosis inhibitor emricasan, necroptosis inhibitor necrostatin-1, and autophagy inhibitor 3-methyladenine (73, 74). Besides, compared with apoptosis-defective (Ripk3^–/–^) mice and necroptosis-defective (Mlkl^–/–^) mice, intraperitoneal injections of Fer-1 (20 mg/kg) followed by DOX in normal mice improved their survival rate remarkably (74). The mechanisms of DOX causing cardiac ferroptosis are as follows (Figure 2 and Table 1):

**TABLE 1:** The main molecular mechanism of DOX-induced ferroptosis in cardiomyocytes.

Experimental model	DOX dose/Route of administration	Findings (mechanisms)	References
**Lipid peroxidation**			
H9C2 cells; Mice	2 μM (*in vitro*) 6 mg/kg, tail vein injection; on days 0, 2, and 4 (*in vivo*)	Mitochondrial GPX4↓	(50)
H9C2 cells; NRVMs	1 μM (H9C2 cells) 2 μM (NRVMs)	Nrf2 (nuclear)↓/GPX4↓	(102, 103)
HL-1 cells; Mice	2 μM (*in vitro*) 1 mg/kg, IP; every other day for 8 times (*in vivo*)	Acot1↓	(69)
H9C2 cells; Mice, rats	5 μM (*in vitro*); 15 mg/kg, IP; for 8 days (*in vivo*)	miR-140-5p ↑/Nrf2 ↓, Sirt2 ↓pathway	(77)
**Iron metabolism**			
H9C2 cells; Rats	2 μM (*in vitro*) 20 mg/kg, IP; a single dose (*in vivo*)	HMGB1 ↓	(73)
Mice	10 mg/kg, IP; a single dose	HO-1 ↑	(74)
H9C2 cells	5 μM DOX	FoxO4 ↑/Enpp2 ↓	(79)
H9C2 cells; Mice	10 μM (*in vitro*) 5 mg/kg, IP; one dose per week for 5 times (*in vivo*)	FXN ↓	(87)
BAEC	0.5 μM	TfR ↑	(88)
AC16 cells	2 μM	METTL14 ↑/KCNQ1OT1 ↑/miR-7-5p ↓/TfR ↑	(89)
H9C2 cells; HL-1 cells	5 μM, 10 μM (H9C2 cells) 5 μM (HL-1 cells)	Ferritin ↑ (especially FTH)	(91, 92)
H9C2 cells; Rats	1 μM (*in vitro*) 2.5 mg/kg, tail vein injection; once a week for 6 weeks (*in vivo*)	Nrf2 (nuclear) ↓/GPX4 ↓, HO-1 ↓, FTH1 ↓, FPN ↑	(105)
NRCMs; Mice	10 μM (*in vitro*) 6 mg/kg, IP; one dose every third day for 4 times OR 10 mg/kg, IP; one dose over 5 days for 3 times (*in vivo*)	ABCB8 ↓	(109)
Rat cardiomyocytes	5 μM	Inhibit Fe mobilization from ferritin	(119)
H9C2 cells; Mice	10 μM (*in vitro*); 5 mg/kg, IP; one dose per week for 5 weeks (*in vivo*)	FXN ↓	(87)
**IREs/IRPs**			
H9C2 cells; Rat primary cardiomyocytes	1, 2.5, 5, and 10 μM (H9C2 cells); 1, 5, 10, and 20 μM (rat primary cardiomyocytes)	Inactivate IRP1 and IRP2	(93, 96)
Mice	15 mg/kg, IP; a single dose	Inactive IRP2/ferritin ↑, TfR1 ↓, unchanged IRP1 activity	(97)

### Doxorubicin and Reactive Oxygen Species

The quinine moiety of DOX received electrons from NADPH oxidase and nitric oxide synthase (NOS) to become semiquinones, which was then accompanied by the ROS production such as O_2_^•–^ and OH^•^ generation (75). In addition, DOX can turn Fe^3+^ into Fe^2+^, thereby aggravating the Fenton reaction to produce more ROS (76). DOX can lead to downregulation of the antioxidant system. Several studies support the view that the levels of antioxidant substances (GPX4, SOD, and GSH) in DOX-treated rats and mice were significantly lower and the content of MDA was significantly higher than in control groups (50, 77, 78). One study suggested that ENPP2 overexpression enhances the expression levels of the ferroptosis-associated gene “GPX4” in H9C2 cells while FoxO4 regulates gene transcription negatively by the suppression of post-transcriptional coding mRNAs. In the H9C2 cells overexpressing ENPP2, DOX-induced increased Fe^2+^ activity, ROS and NOX4 production, while decreasing SLC7A11 and reversing GPX4 and FPN expression (79).

### Doxorubicin and Iron

#### Iron Plays a Pivotal Role in Doxorubicin-Induced Cardiomyopathy

We know that ferroptosis is an iron-dependent lipid peroxidation process, and iron is essential in both the occurrence and development of this process. It is generally believed that excessive iron can aggravate DIC. More than 20 years ago, research indicated that iron overload aggravated DIC (80). DOX reduced the viability of H9C2 cardiomyocytes, while ferric ammonium citrate aggravated it in a concentration-dependent manner (81). Male Sprague Dawley rats fed with iron-rich chow showed significantly higher DOX cardiotoxicity, accompanied with a significant weight loss and severe myocyte injury as evidenced through electron microscopy and light microscopy. However, feeding an iron-rich meal alone did not result in any cardiotoxicity (82). Paradoxically, in cultured H9C2 cardiomyocytes and male C57BL/6 mice, researchers concluded that pretreatment with dextran-iron (125–1,000 μg/mL) in combination with DOX did not potentiate DIC and even prevented some aspects of it (83). Therefore, the regulation of DIC by iron is a complicated process, which may involve the balance between iron dosage, protection, and damage.

The HFE gene encodes HFE protein, which binds to TfR1 and facilitates the uptake of iron-bound to Tf. The elevation of iron concentration in the heart was much more accentuated in DOX-treated HFE^–/–^ mice. Mutations in the HFE gene led to iron overload in cardiomyocytes, which increased the susceptibility of cardiomyocytes to ferroptosis and exacerbated DIC (84). One study concluded that the mutation status of HFE RS1799945 H63D could be used as one of the critical markers to identify patients at high risk for AIC (85). Among survivors of high-risk acute lymphoblastic leukemia in children, patients with the C282Y mutation of the HFE gene had a more severe DIC, which was reflected in higher levels of cardiac troponin-T, lower left ventricular quality and thickness, and worsened left ventricular function in echocardiography (86). These studies on the iron-regulated gene HFE support the view that iron plays a pivotal role in DIC.

#### Changes in Iron Homeostasis Regulatory Proteins

Proteins associated with iron transport are considered significant indicators of cellular iron homeostasis and are mainly involved in cellular iron uptake (TfR1) and storage (ferritin). As for TfR1, most studies believe that DOX could elevate its expression (87–89). A study believed that TfR1 is critical for the DOX-induced increase in iron uptake. After incubation with the specific anti-TfR antibody (12 μg/ml), DOX-induced increase of ^55^Fe uptake in bovine aortic endothelial (BAEC) cells was reversed (88). The mechanism of increased TfR1 could be because DOX inhibited the expression of miR-7-5p by increasing the METTL14-mediated expression of KCNQ1OT1m6A, thus reducing the degradation of TfR1 (89). In AC16 cells, METTL14 knockdown, KCNQ1OT1 silencing, and miR-7-5p mimic attenuated the DOX-induced increase of Fe^2+^ and lipid ROS, and reduced DOX-induced decrease in mtDNA and MMP levels. In METTL14 shRNA mice models, DOX-induced increase in the levels of MDA and 4-HNE were also alleviated (89). However, one study suggested that TfR1 was reduced in heart lysates extracted from DOX-treated mice that received a single i.p. DOX dose (20 mg/kg) as observed in Western-blot and RT-PCR analysis (90). As for ferritin, DOX elevated it, and this change was accompanied by an increase in the level of iron bound to it (90). After being treated with 5 and 10 μM DOX for 24 h, the content of ferritin, especially ferritin heavy chain (FHC), in H9C2 cells increased, and was ROS-dependent. The increase in DOX-induced ferritin was reversed after clearing ROS using N-acetylcysteine (a known ROS scavenger) in H9C2 cells. Interestingly, the increased ferritin induced by DOX protected H9C2 cells from iron toxicity, as demonstrated by increased cell viability in 500, 750, and 1,000 μg/ml FAC measured by MTT assays (91). Similarly, ferritin mRNA and protein levels were also increased in an adult cell line of cardiomyocytes (HL-1) exposed to 5 μM DOX (92). In addition, elevated intracellular iron levels were also associated with high mobility group box 1 (HMGB1)-mediated heme degradation. Compared with the DOX group, the ferroptosis-related indexes (PTGS2, MDA, and 4-HNE) and cardiac injury-related indexes (Anp, Bnp, and Myh7) of the rat heart in the DOX + shHMGB1 group were significantly decreased (73). Therefore, the effect of DOX on iron homeostasis regulatory proteins is a complex process, possibly related to cell lines, drug dosage, and imbalances in protection and injury.

#### Changes in Iron-Responsive Elements/Iron Regulatory Proteins

At present, many studies have shown that DOX can act on IRPs, but the results are numerous. Some studies believed that DOX irreversibly inactivated IRP1 and IRP2. This study believed that the secondary alcohol doxorubicinol (DOXol) and certain products of DOX metabolism, converted cytoplasmic aconitase to the cluster-free IRP1 by removing iron from its catalytic [Fe-S] clusters. This eventually produced a null protein, which could not adapt the levels of TfR1 and ferritin, and IRP2 was inactivated only by DOX related ROS (93). Some scholars thought H_2_O_2_ activated IRP1, leading to the upregulation of TfR1 and cellular iron accumulation, which was considered an important molecular mechanism in DIC (94). One study demonstrated that the IRP1 activity of BAEC cells increased in a dose-dependent manner after being treated with 0.5 μM DOX (88). However, one study found that even exposing DOX-sensitive GLC4 cells to 12.5 μM DOX for 24 h did not change IRP activity. It can be seen that the effect of DOX on cellular IRP activity may be related to cell line and cell resistance (95). However, Juliana et al. believed that the effect of DOX on cardiomyocyte IRP activity was closely related to DOX concentration and incubation time. After 6 h of DOX administration, total IRP1 did not change significantly, but active IRP1 and active IRP2 decreased in a concentration-dependent manner (1 μM DOX, 5 μM DOX, 10 μM DOX, and 20 μM DOX). IRP2, in particular, dropped by more than 50% at 20 μM DOX. After 24 h of administration, while the total IRP1 level decreased, the active IRP1 and active IRP2 did not significantly reduce but even increased. Interestingly, they thought that the free radical scavengers, DOXol, and *cis*-aconitate had little effect on IRP-RNA-binding activity in SK-Mel-28 melanoma cells and cardiomyocytes. The Fe and Cu complexes of anthracyclines altered iron metabolism in cardiomyocytes (96). Gianfranca suggested that DOX had differential effects on the two IPRs, as evidenced by reduced IRP2 activity and unchanged IRP1 activity. The effect of DOX on IRP2 resulted in an upregulation of ferritin gene expression and a decrease in TfR1 expression. Surprisingly, this change reduced the iron content in LIP and protected cardiomyocytes from ferroptosis induced by DOX (97). Furthermore, one study concluded that DOX at low concentrations (≈1 μM) activated IRP1 in cardiomyocytes, while at higher concentrations (>5 μM), it irreversibly inactivated IRP1 in BAEC cells (88). Besides, according to one study, DOX can directly interact with IREs. DOX intercalated double-stranded RNA by recognizing the IREs hairpins located in the 50-UTR of ferritin mRNAs, thereby changing the tertiary structure of the RNA drastically altering the effectiveness of the IREs/IRPs interaction (98). Anyway, DOX could modify the expression of genes involved in iron metabolism by inactivating IRPs binding to IREs (99).

#### Changes in the Nuclear Factor (Erythroid-Derived 2)-Like 2 Signaling Pathway

Under normal physiological conditions, Nrf2 binds to Keap1 in the cytoplasm, and then Nrf2 is degraded by the ubiquitin-proteasome system. In the case of oxidative stress, Nrf2 dissociates from Keap1 and translocates to the nucleus, where it binds to promoter regions (AREs), activates the transcription of a series of downstream genes, and exerts physiological functions (100). Notably, many of the genes associated with ferroptosis are target genes for Nrf2, but DIC-related studies mainly focus on GPX4 and HO-1 genes. Nrf2 up-regulates the expression of GPX4 and has an anti-ferroptosis effect (101–103). Nrf2 can be methylated by the protein arginine methyltransferase 4 (PRMT4), leading to its nuclear restriction and consequently decreased GPX4 expression. PRMT4 aggravated the expression of ferroptosis markers (ROS, MDA, NCO4, and Fe^2+^) in the DOX-induced primary neonatal rat ventricular myocytes and C57BL/6 J mice cardiotoxicity models, and this influence can be mitigated by PRMT4 knockout (103). However, studies have also shown that Nrf2-mediated activation of HO-1 promotes ferroptosis. HO-1 mediates the release of Fe^2+^ from heme, which accumulates in cardiomyocytes and induces ferroptosis (74, 104). Through Nrf2^+/+^ mice, Nrf2^–/–^mice, Znpp (an HO-1 inhibitor), Hemin (an HO-1 activator), DOX has been proven to increase HO-1 by affecting Nrf2 and accelerating the degradation of heme, leading to an increase in non-heme iron and myocardial ferroptosis. An increase in intestinal iron absorption did not accompany this increase of iron content. In this study, DOX was confirmed to be able to upregulate hepatic Hamp1 mRNA to increase hepcidin and reduce FPN degradation (74). In addition, Nrf2 can be activated by deacetylation of SIRT1, and Fisetin activated Nrf2 by up-regulating the expression of SIRT1, leading to the up-regulation of HO-1, FTH1, TfR1, and FPN, and exerting an anti-ferroptosis effect. After being transfected with SIRT1 and Nrf2 siRNA, the anti-ferroptosis effect of Fisetin was abolished in H9C2 cells (105).

### The Role of Mitochondria in Doxorubicin-Induced Cardiomyopathy

#### Mitochondria Is the Major Source of Reactive Oxygen Species

The morphological changes of ferroptosis under the electron microscope were mainly observed in the mitochondria (11). The metabolic activity of mitochondria drives ferroptosis. Mitochondria are the main source of cellular ROS. When electrons are transferred to O_2_, some escape from the ETC and react directly with O_2_ to form O_2_^•–^, a precursor to many other ROS such as OH^•^ and H_2_O_2_ (106). Energy stress inhibits ferroptosis by activating AMPK, while AMPK inactivation promotes ferroptosis (107). ETC complex inhibitors can inhibit ferroptosis, indicating that mitochondria play an important role in ferroptosis, and possibly through the activation of AMPK (108). The mitochondrial TCA cycle is involved in ferroptosis induced by Cys deprivation, for which glutaminolysis is essential. However, mitochondria are not essential for ferroptosis induced by GPX4 inhibition (108).

#### Mitochondria May Be the Site of Iron Accumulation

Doxorubicin can cause cell iron metabolism disorders, but there is much debate about where iron metabolic disorders occur. Some studies have suggested that the site where the DIC occurs is the mitochondria. Compared with cytoplasm, DOX and iron preferentially accumulated in mitochondria (109), especially in mitochondrial cardiolipin (110–112). After incubation with 10 μM DOX, the accumulation of DOX in the mitochondria of neonatal rat cardiomyocytes increased significantly compared with the cytoplasm, and DOX caused a significant increase in mitochondrial iron levels detected by ^55^Fe colorimetric measurement of mitochondrial non-heme iron (109). In the presence of Fe^2+^, DOX induced the activation of the mitochondrial permeability transition pore (113). Tadokoro et al. believed that ferroptosis was triggered by GPX4 deficiency in mitochondria for the following reasons: (1) DOX-induced lipid peroxidation occurred on mitochondria rather than other organelles; and (2) even though the cell viability was improved and lipid peroxidation indexes (MitoPeDPP, MDA) were reduced in both isolated neonatal rat ventricular cardiomyocytes cells transfected with Ad-cytoGPx4-FLAG and Ad-mitoGPx4-FLAG, electron microscopy revealed that mitochondrial GPX4 was almost exclusively localized to mitochondria during this process. In contrast, cytoplasmic GPX4 was transferred to mitochondria (50). Mitochondria-2,2,6,6-tetramethylpiperidin-N-oxyl, a mitochondrial superoxide scavenger, abolished DOX-induced lipid peroxidation and cardiac ferroptosis in DOX-treated mice models. In contrast, the non-mitochondrial targeted version only mildly rescued the DOX-induced effects (74). Dexrazoxane (DXZ) reduced iron in mitochondria. The poor impact of DFO in treating DIC compared with DXZ may be attributed to its inability to penetrate mitochondria and chelate iron in mitochondria specifically (109, 114). The most likely target of free radicals produced by ANTs through redox reactions was cardiolipin, a major phospholipid component of the inner mitochondrial membrane, known to be susceptible to peroxidative injury with abundant PUFA. Iron overload can aggravate the damage of ANTs to the mitochondria of cardiomyocytes (115).

The mechanism of DOX causing myocardial ferroptosis may be related to its effect on mitochondrial iron regulation-related proteins. Compared with wild-type mice, cardiotoxicity was more pronounced in FtMt^–/–^ mice injected intraperitoneally with a single dose (15 mg/kg of body weight) of DOX, as manifested by higher mortality, more morphological changes (incomplete cristae, condensation, and fragmentation of most myofibril), more severe lipid peroxidation, and worse cardiac function (ATP and BNP) (116). In mice, neonatal cardiomyocytes, and H9C2 cardiomyoblasts, DOX led to the reduction of frataxin, a nuclear-encoded mitochondrial protein involved in maintaining mitochondrial iron homeostasis through the ubiquitin-proteasome system. In addition, the mitochondrial iron export protein ABC protein-B8 (ABCB8) is essential for maintaining mitochondrial iron homeostasis. The depletion of ABCB8 led to compromised systolic and diastolic functions, a significant accumulation of ^55^Fe in the mitochondria, and higher lipid peroxidation levels both *in vivo* and *in vitro* (87, 109, 117). The levels of ABCB8 in explanted hearts from patients with end-stage cardiomyopathy were significantly reduced (117). Intriguingly, one study concluded that there was no effect on ABCB8 in the myocardium of the DIC mice models, and the silencing of ABCB8 did not increase the iron content in cultured cardiomyocytes after 30 h of exposure to 2 μM DOX (50).

On the contrary, another study showed that DOX affected more cytosolic than mitochondrial iron metabolism in murine hearts and human HeLa cells, as manifested by alterations in proteins associated with cytoplasmic iron transport proteins (ferritin, TfR1, and hepcidin). In contrast, mitochondrial iron-related proteins (aconitase, succinate-dehydrogenase, and frataxin) appear to be unaffected (90). In addition, Kwok thought that the mechanism of DOX-induced cardiomyocyte ferroptosis was related to lysosomes. It may be that ANTs act on lysosomes to inhibit ferritin degradation, thereby affecting the iron-dependent life activities of cells. However, this study was conducted in SK cells, not cardiomyocytes, so the conclusion is debatable (118). Interestingly, some studies believed that DOX affected neither total cellular Fe content nor total cellular ferritin protein levels (119). Furthermore, the myocardial iron content was not statistically different between the saline and the DOX groups (82). In the presence of NADPH-cytochrome p450 reductase, ANTs underwent redox cycling to generate superoxide, which mediated a slow reductive release of iron from ferritin. More the cardiotoxic ANTs, more the rapid and extensive iron release it led to (120).

### Doxorubicin and Lipid Peroxidation

As mentioned earlier, iron promotes ROS generation through the Fenton and Harber-Weiss reactions (42, 121, 122). PUFA undergoes lipid peroxidation under the action of ROS, which in turn leads to ferroptosis. Furthermore, DOX can also be combined with Fe^3+^ to form the DOX-Fe^3+^ complex (123, 124), which generates the DOX-Fe^2+^ complex through both enzymatic or non-enzymatic reactions. And this DOX-Fe^2+^ complex reacts with oxygen to form O_2_^•–^, which is transformed into OH and H_2_O_2_ through disproportionation reaction (125). H_2_O_2_ can also react with DOX-Fe^2+^ complexes to generate OH^•^ (126). Thus, under the action of the DOX-iron complex, OH and O_2_, PUFA undergoes lipid peroxidation. In addition, even without free Fe^3+^ and Fe^2+^, DOX can extract Fe^3+^ directly from ferritin to form DOX-Fe^3+^ complexes, resulting in lipid peroxidation (127). In this process, it is not the free Fe^3+^, free Fe^2+^, or DOX-Fe^3+^ iron complexes, but the DOX-Fe^2+^ complexes that induce ferroptosis in cardiomyocytes. The use of specific Fe^2+^ chelators like Mito-FerroGreen and bathophenanthroline effectively attenuate cardiomyocyte lipid peroxidation, measured using C11-BODIPY 581/591 and MDA (50, 124). It should be noted that lipid peroxidation is not the only pathological change brought about by ferroptosis. Oxidative stress, apoptosis, necrosis, and other forms of RCD share characteristics of lipid peroxidation. So, if we want to prove that ferroptosis is DIC’s mechanism, we must measure lipid peroxidation directly (56).

## Prevention and Treatment of Anthracycline-Induced Cardiotoxicity Based on Ferroptosis

**FIGURE 3:**
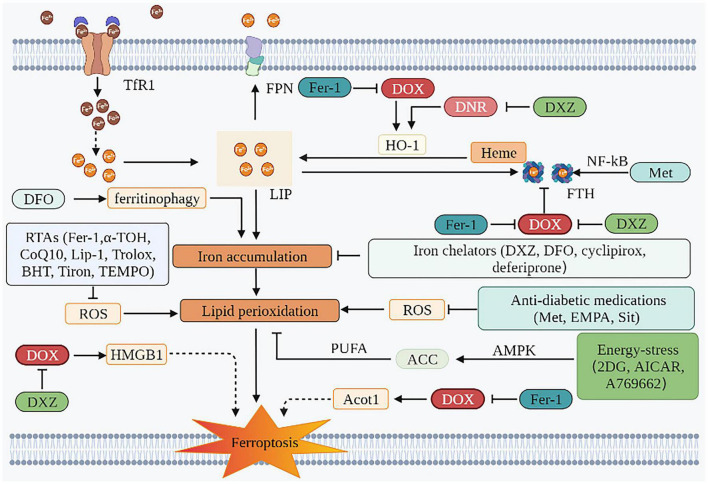
Prevention and SOD treatment of AIC based on ferroptosis. The mechanism of preventing ferroptosis of cardiomyocytes is mainly in two aspects. One is to inhibit iron accumulation, and the other is to inhibit lipid peroxidation. Iron chelators can play a role through the former. The effects of RTAs, anti-diabetic medications, and energy-stress inducers are mainly attributed to the latter. TfR1, transferrin receptor 1; FPN, ferroportin; DOX, doxorubicin; DNR, daunorubicin; LIP, labile iron pool; HO-1, heme oxygenase 1; ROS, reactive oxygen species; Fer-1, ferrostatin-1; DFO, deferoxamine; DXZ, dexrazoxane; PUFA, polyunsaturated fatty acids; ACC, acetyl-CoA carboxylase; AMPK, AMP-activated protein kinase; EMPA, empagliflozin; Sit, sitagliptin; 2DG, 2-deoxy-d-glucose; AICAR, 5-aminoimidazole-4-carboxamide ribonucleotide; HMGB1, high mobility group box 1; HO-1, heme oxygenase 1; FTH, ferritin heavy chain; NF-κB, nuclear factor-kappa B; α-TOH, α-tocopherol; CoQ10, coenzyme Q10; Lip-1, liproxstatin-1; BHT, butylated hydroxytoluene; Acot1, acyl-CoA thioesterase 1; TEMPO, 2,2,6,6-tetramethylpiperidin-N-oxyl; Met, metformin.

Evaluations performed before the initiation of anticancer therapy in patients without significant CVDs should be regarded as the primary prevention strategy (128). An appropriate cancer treatment and anti-cardiotoxicity prevention and treatment strategies should be selected after a comprehensive discussion by a multidisciplinary team of cardiovascular, oncology and hematology experts, especially to balance the effects of drugs after cancer treatment regimens and the risk of specific CVDs in all aspects (129). Commonly used drugs and their mechanism of action are described as follows (Figure 3 and Table 2):

**TABLE 2:** The therapeutic strategies against ferroptosis in DIC.

Agent	Study design	DOX administration	Agent dose	Mechanism	Parameters	References
DXZ	*In vivo* (mice)	3 mg/kg, IP; once per week for 6 weeks	30 mg/kg, IP; once per week for 6 weeks	Chelating iron	ECV ↓, GCS ↑, GLS ↑, LVEF ↑, T2 Avg ↓	(130)
DXZ	*In vivo* (mice)	6 mg/kg, tail vein injection; on days 0, 2, and 4	1,000 μM	Mitochondrial GPX4 ↑	Mitochondrial lipid peroxidation ↓, MDA ↓, cell survival rate ↑, mitochondrial iron ↓	(50)
DXZ	*In vivo* (rats)	20 mg/kg, IP; a single dose	NA	HMGB1 ↓	PTGS2 ↓, MDA ↓, Anp ↓, Bnp ↓, Myh7 ↓, LVEF ↑, LVFS ↑, cardiac heme ↑, serum heme ↑, non-heme iron ↓, Tfrc ↑, FTH1 ↓	(73)
DXZ	*In vitro* (H9C2 cells)	2 μM	100 μM/L	HMGB1 ↓	Cell viability ↑, PTGS2 ↓, MDA ↓, LDH ↓, Fe^2+^ ↓, GPX4 ↑, FTH1 ↑	(73)
DFO	*In vivo* (mitochondria from rat hepatocytes and cardiomyocytes)	50 μM	250 μM	Protect MPTP	Ca^2+^-induced MPTP activation ↓, MMP ↑, SDH ↑	(113)
Fer-1	*In vitro* (H9C2 cells)	2 μM	50 μM	Mitochondrial GPX4 ↑	Mitochondrial lipid peroxidation ↓, MDA ↓, cell survival rate ↑, mitochondrial iron ↓	(50)
Fer-1	*In vivo* (mice)	10 mg/kg, IP; a single dose	1 mg/kg, IP; a single dose before DOX treatment	HO-1 ↓	Collagen ↓, Anp ↓, Bnp ↓, Myh7 ↓, EF ↑, FS ↑, heart rate ↑, PEox ↓, dioxide PEox ↓, trioxide PEox ↓	(74)
Fer-1	*In vivo* (mice)	15 mg/kg, IP at day 1 and 10 mg/kg IP at day 8	1 mg/kg, IP; every other day for 8 times	Acot1 ↑	Survival rate ↑, EF ↑, FS ↑, LVIDd ↓, LVIDs ↓, collagen area ↓, PTGS2 ↓, MDA ↓, mitochondrial morphological changes ↓	(69)
Fer-1	*In vitro* (HL-1 cells)	2 μM	10 μM	Acot1 ↑	Cell viability ↑, GSH ↓, PTGS2 ↓, lipid ROS ↓	(69)
Fer-1	*In vivo* (rats)	20 mg/kg, IP; a single dose	1 mg/kg, IP; a single dose before DOX treatment	HMGB1 ↓	PTGS2 ↓, MDA ↓, Anp ↓, Bnp ↓, Myh7 ↓, LVEF ↑, LVFS ↑	(73)
Fer-1	*In vitro* (H9C2 cells)	2 μM	10 μmol/L	HMGB1 ↓	Cell viability ↑, PTGS2 ↓, MDA ↓, LDH ↓, Fe^2+^ ↓, GPX4 ↑, FTH1 ↑	(73)
EMPA	*In vitro* (HL-1 cells)	100 nM	10, 50, and 500 nM	NLRP3/MyD88-related pathway ↓	Cell viability ↑, ROS ↓, MDA ↓, 4-HNA **↓**, IL-1β↓, IL-6 ↓, IL-8 ↓, leukotrienes B4 ↓	(156)
EMPA	*In vivo* (mice)	2.17 mg/kg/day, IP; for 7 days	10 mg/kg/day, oral gavage; for 10 days	NLRP3/MyD88-related pathway ↓	MitoPeDPP ↓, MDA ↓, xanthine oxidase ↓, IL-1β↓, IL-6 ↓, IL-8 ↓, MyD88 ↓, NLRP3 ↓, EF ↑, FS ↑, fibrosis ↓	(156)
Met	*In vitro* (HL-1 cells)	5 μM	4 mM	FHC ↑, NF-κB ↑	Cell viability ↑, ROS ↓, CAT ↑, Gpx3 ↑, SOD ↑, free iron ↓ Complex I activity ↑, ATP ↑, loss of ΔΨm ↓	(92, 160)

### Dexrazoxane

Dexrazoxane is the only formally preventive drug approved by the FDA. For patients planning to receive high-dose of ANT therapy, DXZ is recommended (2). DXZ is traditionally known as an iron chelator. Under the action of the iron-ANT complex, the ring of DXZ was opened and hydrolyzed to ADR-925. This presumably exerted its cardioprotective effects by either binding freely or loosely to iron or iron complexed with DOX, thus preventing or reducing site-specific oxygen radical production that damages cellular components (130). In addition, DOX-induced cardiac ferroptosis in rats was observed to be mediated by the upregulation of HMGB1, and correspondingly ferroptosis was inhibited when shHMGB1 was used. DXZ reversed DOX-induced elevation of HMGB1. DXZ also modulated iron metabolism-related protein levels in cardiac myocytes and reversed the upregulation of HO-1 induced by daunorubicin. It therefore inhibited the conversion of heme iron to non-heme iron, which reduced the Fe^2+^ content in the LIP in cardiac myocytes (131). In addition, this study also showed that DXZ reversed the DOX-induced decrease of FTH1 protein in the H9C2 cells (73). However, this view has been greatly challenged. An increasing number of studies believe that DXZ plays a protective role in the heart mainly because it inhibited DOX-mediated damage of cardiomyocyte topoisomerase IIβ (132, 133). Also, in one study, the chelating metabolite ADR-925 therapy on neonatal ventricular myocytes receiving was neither able to mitigate AIC, nor did it significantly impact daunorubicin-induced mortality, blood congestion, and biochemical and functional markers of cardiac dysfunction in a chronic rabbit model *in vivo* (132). Although the mechanism of DXZ chelating iron in mitochondria does not depend on the topoisomerase IIβ pathway (109), the contribution of DXZ in regulating the cardioprotective effect against ferroptosis is not apparent. Besides, DXZ has been shown to inhibit DOX-induced cardiomyocyte necrosis and apoptosis through several alternate mechanisms (134–136). Therefore, the cardiomyocyte protective effect exerted by DXZ is not achieved only through the inhibition of ferroptosis as it is not a simple, specific ferroptosis inhibitor.

Although DXZ is generally considered to reduce AIC and is recommended as the only approved cardioprotective agent (137, 138), the clinical use of DXZ is encountering various challenges. Firstly, there are concerns that DXZ may increase the risk of acute myeloid leukemia and secondary solid tumors in children (139–141). Secondly, the effectiveness of DXZ is also being questioned. One study found that the preventive use of DXZ before high-dose DOX in eight sarcoma patients did not reduce their cardiotoxicity satisfactorily. There were six patients with high-sensitivity troponin T levels exceeding 10 ng/ml, four patients with LVEF that decreased by more than 5%, and three patients with global longitudinal peak systolic strain changed by more than 15% (142). Finally, the European Society of Cardiology only recommends DXZ for patients with advanced or metastatic breast cancer receiving cumulative doses of DOX over 300 mg/m^2^ (2). In spite of this, DIC development is not a dose-dependent response, and in our clinical work, we have found that some patients develop DIC when treated with small doses of anthracyclines.

### Deferoxamine

Deferoxamine is a widely used iron chelator that can chelate excess intracellular iron, thereby reducing DOX-induced ferroptosis in cardiomyocytes. In addition, DFO can also be regarded as a protective agent for mitochondrial permeability transition pore, as it weakens the opening of calcium-dependent pores induced by iron and iron-DOX complexes and reduces the uptake of Fe^2+^ in mitochondria, thus protecting mitochondrial function (113). Intriguingly, DFO was demonstrated to aggravate ferroptosis by inducing ferritinophagy, leading to the accumulation of iron and ROS (10, 143, 144). The effect of DFO in protecting cardiomyocytes from DIC is still contentious. In this study, the cardioprotective benefit of DFO requires a strict dose, and a slight deviation from it would diminish this effect (145). Besides, DFO failed to reverse myocardial damage in a well-established spontaneously hypertensive rat models of chronic ANT cardiomyopathy (146). Moreover, DFO’s side effects, such as hypotension and renal insufficiency, limit its clinical application (147).

### Ferrostatin-1

As a radical-trapping antioxidant (RTA), Fer-1 attenuates lipid peroxidation by decreasing erastin-induced accumulation of cytosolic and lipid ROS, consequently inhibiting ferroptosis (11). This is primarily due to its powerful chain-carrying peroxyl trapping ability (148). The experimental results have shown that Fer-1 could reduce the lipid peroxides labeled with MDA and MitoPeDPP (50). In addition, the mechanisms by which Fer-1 protects the heart from DOX-induced ferroptosis could be describes as follows: Fer-1 inhibited DOX-induced elevation of non-heme iron in cardiomyocytes by downregulating Nrf2/HO-1, thereby inhibiting ferroptosis (74). In an alternate way, DOX downregulated the Acyl-CoA thioesterase 1 gene, causing alterations in the composition of free fatty acids in mitochondrial membranes, particularly in the proportion of C22:6N3, leading to ferroptosis. Fer-1 inhibited DOX downregulation of this gene which reduced the sensitivity of cardiomyocytes to ferroptosis induced by DOX (69). Besides, Fer-1 increased the expression of FTH1 protein to increase iron in storage, resulting in the reduction of Fe^2+^ in LIP (73).

### Other Iron Chelators and Radical-Trapping Antioxidants

In addition to Fer-1, parallel experiments in GPX4 gene-deficient mouse embryonic fibroblast ferroptosis model and extracellular high concentration glutamate-induced ferroptosis model have shown that other RTAs (liproxstatin-1 and α-TOH) can also improve cell survival rate. In HEK-293 cells, the mechanism by which Fer-1, liproxstatin-1, and α-TOH inhibit lipid hydroperoxides and bring about ferroptosis was demonstrated to be achieved by capturing chain-carrying peroxyl radicals, rather than inhibiting LOXs or restoring GSH levels (148–150). However, in a striatal cell model, the mechanism of action of α-TOH against ferroptosis was proved to be associated with LOX. Its endogenous metabolite, α-tocopherol hydroquinone, inhibited 15-LOX activity by reducing its non-heme Fe^3+^ center to the inactive Fe^2+^, thereby inhibiting lipid peroxidation (151). Other antioxidants (Trolox, butylated hydroxytoluene, Tiron, and TEMPO) have less inhibitory effects on erastin-induced ferroptosis than Fer-1 and this could be attributed to Fer-1 containing an aromatic amine (11). Another RTA, CoQ10, was shown to inhibit ferroptosis by inhibiting the propagation of lipid peroxides. Inhibition of CoQ10 synthesis with 4-chlorobenzoic acid or knockout of COQ2, an enzyme required for CoQ10 synthesis, can increase the sensitivity of cells to RSL3-induced ferroptosis *in vitro* (152, 153). Other iron chelators such as ciclopirox and deferiprone also alleviate iron-dependent lipid peroxidation by depriving iron (12).

Although many experiments have demonstrated that ferroptosis could be attenuated by inhibiting lipid peroxidation and iron accumulation, studies using DOX-induced cardiomyopathy models are scarce, and the efficiency and safety of these drugs against ferroptosis are still questionable (56).

### Anti-diabetic Medications

According to recent research, DIC was also associated with insulin signaling imbalance and cardiac insulin resistance (154, 155). Metformin, empagliflozin, and sitagliptin have been shown to reduce ferroptosis triggering lipid peroxidation in the mitochondria and cytoplasm of cardiomyocytes (156–158). Metformin and sitagliptin have been shown to attenuate myocardial lipid peroxidation in rats by reversing the DOX-induced decrease in GSH (159). In cardiomyocytes (HL-1 cell line) exposed to DOX and C57Bl/6 mice treated with DOX, empagliflozin reduced lipid peroxidation levels, decreased cardiomyocyte fibrosis, inhibited cardiomyocyte inflammation, increased ejection fraction percentage (% EF) and fractional shortening percentage (% FS), improved cardiac function, and protected cardiomyocytes from ferroptosis (156). Metformin also activated nuclear factor-kappa B, thereby increasing FTH and reducing iron accumulation in LIP, thus protecting adult mouse cardiomyocytes from DIC (92, 160).

### Energy-Stress Inducers

In immortalized mouse embryonic fibroblasts (MEFs), the energy-stress inducers (2-deoxy-D-glucose, 5-aminoimidazole-4-carboxamide ribonucleotide, and A769662) were demonstrated to activate AMPK. This further inhibited acetyl-CoA carboxylase, and in turn palmitic acid (C16:0), thereby reducing the synthesis of PUFA, all of which suppressed ferroptosis (107).

In conclusion, anti-DIC therapy based on these ferroptosis triggering mechanisms mainly include two aspects: iron chelation and antioxidant treatment. Notably, human induced pluripotent stem cell-derived cardiomyocytes (hiPSC-CMs) have a great potential in predicting patient susceptibility to DIC. Furthermore, the human-derived DOX cardiomyocyte injury model established by hiPSC-CMs overcomes the species differences of current research models and can be accurately used to understand the mechanism of ferroptosis in DIC (161).

## Conclusion and Perspectives

In summary, ferroptosis has been demonstrated by several studies to mediate the occurrence of AIC. In fact, DOX can increase the ROS content and affect iron metabolism in cardiomyocytes by acting on iron homeostasis regulatory proteins, such as, IREs/IRPs, and Nrf2/HO-1, resulting in the accumulation of lipid peroxides, thereby inducing ferroptosis. Mitochondria are the main organelle for inducing ferroptosis in cardiomyocytes. With further research, inhibition of ferroptosis could act as an effective strategy in both prevention and treatment of AIC. This would require screening for potential drugs that inhibit ferroptosis forcefully in cardiomyocytes or developing novel ferroptosis inhibitors and will surely benefit cancer patients with heart diseases as well as patients with high cardiovascular risk stratification.

## Author Contributions

YX and SS designed this study. GZ wrote the first draft of this manuscript and created the figures. CY and XS searched the literature. JZ and XY participated in discussions and improved pictures related to the manuscript. PG, GV, GL, NA, CL, WS, HC, and MW critically revised and approved the final manuscript. All authors contributed to the article and approved the submitted version.

## Conflict of Interest

The authors declare that the research was conducted in the absence of any commercial or financial relationships that could be construed as a potential conflict of interest.

## Publisher’s Note

All claims expressed in this article are solely those of the authors and do not necessarily represent those of their affiliated organizations, or those of the publisher, the editors and the reviewers. Any product that may be evaluated in this article, or claim that may be made by its manufacturer, is not guaranteed or endorsed by the publisher.

## Abbreviations

ANT: anthracyclines
RCD: regulated cell death
ROS: reactive oxygen species
PUFA: polyunsaturated fatty acids
GSH: glutathione
GPX4: glutathione peroxidase 4
FSP1: ferroptosis suppressor protein 1
CoQ10: coenzyme Q10
NADPH: nicotinamide adenine dinucleotide phosphate
GCH1: GTP cyclohydrolase-1
BH4: tetrahydrobiopterin
AIC: anthracycline-induced cardiotoxicity
DIC: doxorubicin-induced cardiomyopathy
DOX: doxorubicin
CVDs: cardiovascular diseases
DFO: deferoxamine
Fe^3+^: ferric iron
Tf: transferrin
TfR1: transferrin receptor 1
Fe^2+^: ferrous iron
DMT1: divalent metal transporter 1
FPN: ferroportin
LIP: labile iron pool
LOX: lipoxygenase
FTH: ferritin heavy chain
IRP: iron regulatory protein
IREs: iron-responsive elements
HO-1: heme oxygenase 1
O2^•–^: active oxygen
OH^•^: hydroxyl radical
PL^•^: phospholipid radical
PLOO^•^: phospholipid peroxyl radical
PLOOH: phospholipid hydroperoxide
MDA: malondialdehyde
Cys: cysteine
Nrf2: nuclear factor (erythroid-derived 2)-like 2
AMPK: AMP-activated protein kinase
HMGB1: high mobility group box 1
DXZ: dexrazoxane
ABCB8: ABC protein-B8
RTA: radical-trapping antioxidant
α-TOH: α-tocopherol
Fer-1: ferrostatin-1
SOD: superoxide dismutase
Trx: thioredoxin
TrxR: thioredoxin reductase
HETE: hydroxyeicosatetraenoic acid
PE: phosphatidylethanolamine
PRMT4: protein arginine methyltransferase 4.
